# Transmural cross-sectional findings and bowel damage assessment in preclinical Crohn’s disease: a case-control study

**DOI:** 10.1007/s00384-024-04660-5

**Published:** 2024-06-14

**Authors:** Iago Rodríguez-Lago, Marta Aduna, Patricia Ramírez de la Piscina, Olga Merino, Juan Carrascosa, Rebeca Higuera, Ainara Maíz, Eva Zapata, José Luis Cabriada, Manuel Barreiro-de Acosta

**Affiliations:** 1 IBD Unit, Gastroenterology Department, Hospital Universitario de Galdakao-Usansolo, Galdakao, Spain; 2 Biobizkaia Health Research Institute, Galdakao, Spain; 3 School of Medicine, Universidad de Deusto, Bilbao, Spain; 4 OSATEK, Galdakao, Spain; 5 Gastroenterology Department, Hospital Universitario Araba, Vitoria, Spain; 6 Gastroenterology Department, Hospital Universitario de Cruces, Barakaldo, Spain; 7 Gastroenterology Department, Hospital de Zumárraga, Zumárraga, Spain; 8 Gastroenterology Department, Hospital San Eloy, Barakaldo, Spain; 9 Gastroenterology Department, Hospital Universitario Donostia, Donostia, Spain; 10 Gastroenterology Department, Hospital de Mendaro, Mendaro, Spain; 11 Gastroenterology Department, Hospital Clínico Universitario de Santiago, Universidad de Santiago de Compostela, Santiago de Compostela, Spain

**Keywords:** Bowel damage, Crohn’s disease, Diagnosis, Early, Preclinical

## Abstract

**Purpose:**

Crohn’s disease (CD) is a progressive disorder leading to cumulative bowel damage. The Lémann index is a validated tool that can help in monitoring the progression of the disease and evaluating the effectiveness of different therapies. Our aim was to describe the main radiological findings in incidentally diagnosed CD and to evaluate bowel damage in this subgroup compared to patients diagnosed at later stages.

**Methods:**

Patients with an incidental diagnosis of CD during the colorectal cancer screening program were compared to controls with a CD cohort diagnosed after symptomatic onset and matched 1:1 by disease extent. All cross-sectional examinations were centrally read, performing a descriptive analysis of the main findings and calculation of Lémann index.

**Results:**

Thirty-eight patients were included: 19 with preclinical CD (median age 55 years (IQR, 54–62), 53% male, 74% non-smokers; 74% B1 and 26% B2) and 19 matched-controls with symptomatic CD. In those with preclinical CD, the most frequent transmural findings on MRE were contrast enhancement (79%), wall thickening (79%), followed by lymphadenopathy (68%), edema (42%), and increased vascularity (42%). Among those with strictures, controls showed a higher rate of preestenotic dilation (100% vs. 0%, *p* = 0.01). Bowel damage assessment revealed no statistically significant differences in the Lémann index between preclinical CD and controls (*p* = 0.95). A statistically significant higher score in the colonic/rectum score was observed (*p* = 0.014).

**Conclusion:**

Patients with preclinical CD demonstrate similar radiological findings and degree of bowel damage as new-onset symptomatic CD.

## Introduction

Crohn’s disease (CD) is a chronic inflammatory bowel disease with an increasing worldwide prevalence [1]. While the exact causes of CD are still unknown, it is considered to be the consequence of a multifactorial interaction between certain genetic and environmental factors, including also the microbiome [2]. CD also involves a progressive inflammatory disorder that leads to disabling symptoms [3]. Still, around one in five patients demonstrate complicated lesions (e.g., strictures, fistula, abscess, perianal disease) upon diagnosis [4, 5], and this is expected to be the consequence of a prolonged time interval between the disease onset and the diagnosis, where subclinical inflammatory pathways have been described to be present for a long time [5, 6]. More importantly, since the diagnosis and onwards, the proportion of patients with complicated phenotypes progressively increases over time, thus highlighting the importance of early identification of subjects at higher risk of developing the disease or with early lesions [4].

The complete pathophysiology of CD is still not fully understood, but most recent data suggest that an altered immune response and even mucosal lesions can be present even years before the diagnosis, similar to what has been described in other immune-mediated diseases like rheumatoid arthritis, systemic lupus erythematosus, or diabetes mellitus type 1 [7]. The detection of mucosal abnormalities in otherwise healthy subjects, particularly in the context of colorectal cancer screening programs, has also unveiled a new subgroup of patients where subclinical lesions could be better characterized [8–10]. The prevalence of these findings within population-based screening programs is approximately 0.35% [8], and up to 58% will develop symptomatic disease after a median of 7 years [11].

Due to this progressive course, cross-sectional imaging evaluation is essential during the initial workup and follow-up of patients with CD [12]. The most recent ECCO-ESGAR guidelines recommend that all patients with newly-diagnosed CD should undergo an evaluation of the small bowel [12]. However, there is no clear recommendation about the best technique (i.e., ultrasound, CT scan, capsule endoscopy, or magnetic resonance enterography (MRE)) in this context. Accumulating evidence supports that MRE seems to be superior to intestinal ultrasound as it is more accurate in defining CD extent and detecting certain complications like fistulas [13]. Apart from local expertise and availability, the rest of the techniques have evident limitations when compared to MRE, including the exposure to radiation (e.g., CT scan) or the evaluation limited to the mucosa (e.g., capsule endoscopy).

Providing the importance of obtaining an overall quantification of bowel damage in CD, a team of international experts developed an index defined as the Lémann index (LI) [14, 15]. Importantly, when assessed by this tool, a significant proportion of patients already demonstrate bowel damage at diagnosis, and this has been linked to worse outcomes during follow-up [16, 17]. Hence, as there is a disconnection between symptoms and more objective examinations, the LI has been shown as a promising prognostic and monitoring tool for CD. While further research is needed to evaluate how the LI can be incorporated into clinical practice and to determine its full potential, it represents an attractive method for improving the individualized assessment and management of this debilitating condition. Therefore, our aim was to describe the most frequent cross-sectional findings in patients with an incidental diagnosis of CD and to evaluate the progression of bowel damage from a preclinical stage until the symptomatic onset of the disease.

## Materials and methods

We performed a multicentric, retrospective, case-control study including all asymptomatic subjects with a diagnosis of CD during the colorectal cancer screening program at seven hospitals in the Basque Country region (Spain; https://www.osakidetza.euskadi.eus/enfermedad-cancer/-/programa-cribado-cancer-colorrectal/) with a target population of 624,471 subjects (Eustat 2020; https://www.eustat.eus/indice.html). The Basque colorectal cancer screening program invites all persons between 50 and 69 years to perform a fecal immunochemical test, followed by a complete colonoscopy in those with a positive result (cut-off 20 µg Hb/g). In this study, we included all patients with a combination of clinical, endoscopic, and histologic data, requiring the presence of chronic infiltrate and absence of any enteropathogen or alternative diagnosis, all of them suggesting a diagnosis of CD [18, 19] and following the same criteria as in our previous reports [8, 9, 11]. The screening program refers all subjects with abnormal findings suggestive of inflammatory bowel disease to the Gastroenterology clinic, where alternative diagnosis is systematically ruled out. During follow-up at the IBD Clinic, all new symptoms and the date of onset were registered.

Patients were compared 1:1 to matched-controls by disease extent (according to Montreal classification [20]) from an inception cohort of patients with CD diagnosed after symptomatic onset of the disease. Both cases and controls were on regular follow-up at the IBD Clinic, and only patients with both MRE and colonoscopy within the first year after diagnosis were considered for this study. All cross-sectional examinations were centrally read by one experienced radiologist (M.A.). We compiled information about transmural (including contrast enhancement, wall thickening, ulceration, edema, pseudopolyps, fat infiltration, strictures, and preestenotic dilation) and extraintestinal findings (including lymphadenopathy, increased vascularity, fat proliferation, bowel wall stratification, abscess or phlegmon, fistula, and free fluid). In addition, the same trained radiologist assessed bowel damage through LI scoring according to its initial description and validation [14, 15]. This index is calculated by dividing the digestive tract into four organs: upper tract, small bowel, colon/rectum, and anus. Each organ is then divided into segments, and for each segment, information on previous surgical procedures is retrieved, and strictures and penetrating lesions are also identified and scored according to their maximal severity (grades 1–3). A segmental damage evaluation ranging from 0.0 to 10.0 (complete resection) is then provided from the standardized cumulative damage evaluations, and an overall organ resection-free cumulative damage evaluation is finally calculated from the sum of segmental damages [15]. Patients with incomplete clinical or radiological data were excluded from the analysis.

This study was approved by the local Ethics Committee; informed consent was provided before any study procedure, and it was conducted under the Declaration of Helsinki. Descriptive statistics were used by using median and interquartile ranges. Comparisons between groups were performed by chi-square tests and Mann-Whitney analysis.

## Results

### Patient characteristics

A total of 38 patients were included: 19 cases with preclinical CD and 19 matched-controls. Their main characteristics are summarized in Table 1. Notably, CD location was described as L1 in 53%, L2 in 21%, and L3 in 26%, with no patients showing upper tract involvement. CD behavior was numerically different between cases and controls, with a higher proportion of patients showing an inflammatory behavior among cases (B1 according to Montreal classification; 74% vs. 58%), while a higher number of controls showed complicated disease (either B2 or B3, Table 1).

**Table 1:** Patient characteristics

	Preclinical CD (*N* = 19)	Controls (*N* = 19)	*p* value
Age, years			
Median, IQR	55 (IQR, 54–62)	49 (IQR, 45–58)	0.60
Mean, SD	57 (4.9)	49 (14.6)	
Sex, male	10 (53%)	8 (42%)	0.75
Smoking habits			
Never	14 (74%)	10 (53%)	0.35
Former	3 (16%)	4 (21%)	
Active	2 (10%)	5 (26%)	
CD location			
L1	10 (53%)	10 (53%)	0.66
L2	4 (21%)	4 (21%)	
L3	5 (26%)	5 (26%)	
L4	0	0	
CD behavior			
B1	14 (74%)	11 (58%)	0.12
B2	5 (26%)	7 (37%)	
B3	-	1 (5%)	
Perianal disease	-	2 (11%)	0.49

*CD* Crohn’s disease, *IQR* interquartile range, *SD* standard deviation

At the moment of performing MRE, 32% of controls were on mesalazine, 26% on systemic or low-bioavailability steroids (or exposed in the past 3 months), and 5% on anti-TNF biologics. However, the proportion of patients receiving biologics or small molecules increased up to 53% mainly based on MRE findings. After a median follow-up of 102 months (IQR, 57–120), 58% of cases (*N* = 11) developed symptoms emerging a median of 10 months (IQR, 5–17) following the diagnosis. Diarrhea was the most frequently reported symptom (8/11, 73%), followed by rectal bleeding (3/11, 27%) and less frequently abdominal pain (2/11, 18%) or weight loss (1/11, 9%). One patient (9%) developed a small bowel obstruction, and another was diagnosed of erythema nodosum (associated with gastrointestinal symptoms).

### Cross-sectional imaging findings

The most frequent transmural findings on MRE in patients with preclinical CD were contrast enhancement and wall thickening (79% each), followed by edema (42%), while among extraintestinal findings, they were lymphadenopathy (68%) and increased vascularity (42%) (Table 2). Among those with strictures, controls demonstrated a higher rate of preestenotic dilation as compared to cases (100% vs. 0%, respectively; *p* = 0.01). There was no association between any of these findings and the risk of developing symptomatic disease during follow-up among cases.

**Table 2:** Cross-sectional findings on patients and controls

	Preclinical CD (*N* = 19)	Controls (*N* = 19)	*p* value
Transmural findings			
Contrast enhancement	15 (79%)	15 (79%)	1.0
Wall thickening	15 (79%)	16 (84%)	1.0
Ulceration	5 (26%)	9 (47%)	0.31
Edema	8 (42%)	9 (47%)	1.0
Pseudopolyps	2 (11%)	1 (5%)	1.0
Fat infiltration	6 (32%)	3 (16%)	0.45
Stricture	5 (26%)	7 (37%)	0.73
Preestenotic dilation^1^	0 (0%)	7 (100%)	0.01
Extraintestinal findings			
Lymphadenopathy	13 (68%)	14 (74%)	1.0
Increased vascularity	8 (42%)	9 (47%	1.0
Fat proliferation	7 (37%)	9 (47%)	0.74
Bowel wall stratification	5 (26%)	3 (16%)	0.69
Abscess of phlegmon	-	1 (5%)	1.0
Fistula	-	1 (5%)	1.0
Free fluid	1 (5%)	3 (16%)	0.60

*CD* Crohn’s disease

^1^Among patients and controls with strictures

### Lémann index

**Fig. 1:**
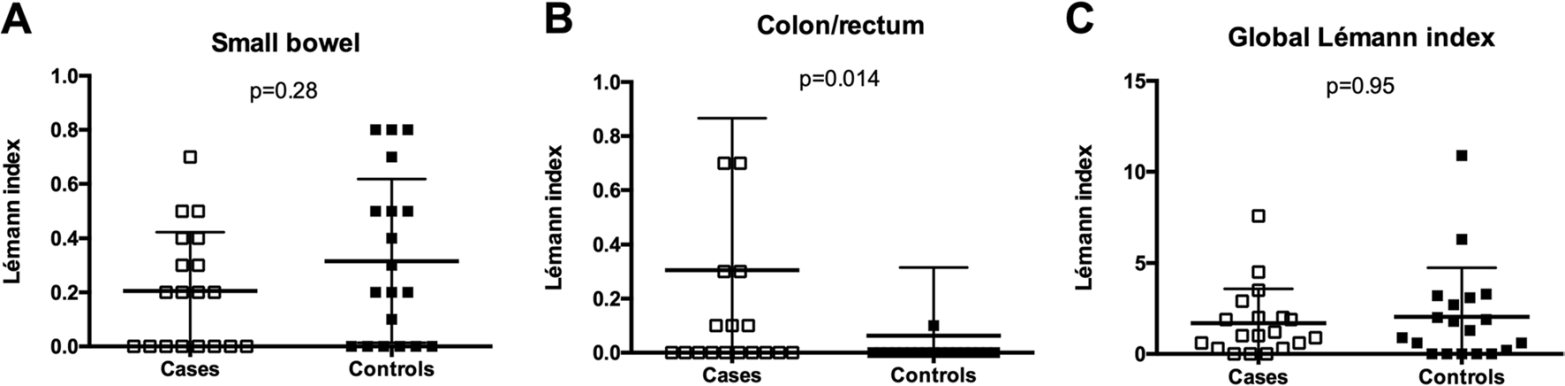
Lémann index among patients and controls, including scores from the small bowel (**A**), colon/rectum (**B**), and global (**C**)

**Fig. 2:**
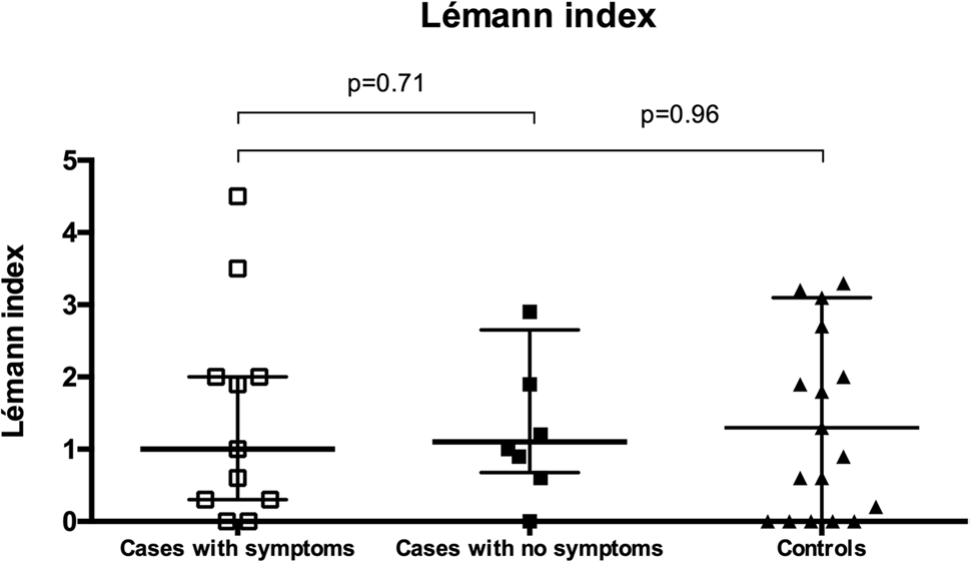
Comparison of bowel damage between cases who developed symptoms vs. those who remained asymptomatic during follow-up and with controls

Bowel damage assessment revealed no statistically significant differences in the LI between preclinical CD patients and controls (median LI 1.0 (IQR, 0.3–2.0) vs. 1.3 (IQR, 0.0–3.1), respectively; *p* = 0.95; Fig. 1). However, we observed that cases had statistically significant higher scores in the colonic/rectum score than controls (*p* = 0.014; Fig. 1). No differences were found in the remaining subscores. Further comparisons were performed, and we observed no differences in the LI between preclinical CD patients who subsequently developed symptoms (median LI 1.0 (IQR, 0.3–2.0)) and those who did not (median 1.1 (IQR, 0.68–2.65), respectively, *p* = 0.71) or with controls (median 1.3 (IQR, 0.0–3.1), *p* = 0.96) (Fig. 2).

## Discussion

In our study, we have described that patients with preclinical CD can already demonstrate a certain degree of bowel damage, and this is comparable to patients who have developed symptomatic disease. A detailed description of the initial cross-sectional imaging findings is also provided, with contrast enhancement, wall thickening, and edema or lymphadenopathy and increased vascularity as the most frequent transmural or extraintestinal signs, respectively. In addition, strictures observed after the symptomatic onset of the disease showed a higher proportion of signs of chronicity than those observed during the preclinical phase.

Due to the need of a scoring system that provides a comprehensive evaluation of the whole gastrointestinal tract and its structural damage, the LI was developed as a promising tool able to evaluate complications such as fistulas, abscesses, strictures, and/or previous surgical resections, therefore being able to measure disease progression over time [14, 15]. Observational studies have demonstrated that LI progressively increases since diagnosis [14], with median scores of 2.3 (IQR, 1.2–3.9) at first evaluation, 3.5 (IQR, 1.2–8.6) at 2 to 5 years after diagnosis, and 8.3 (IQR, 1.2–12.1) at 5 to 10 years after diagnosis [16]. It has also been shown as a prognostic marker, as higher scores at diagnosis predict the need of surgery during the first year [21], so it has been suggested that these cases should be proactively treated and monitored carefully to prevent progressive bowel damage [16, 17, 22]. Similar observations have been done even in patients considered as early CD (< 24 months disease duration with no previous medical therapy or surgery), in whom any relative increase on LI was associated with a worse prognosis [17]. Hence, the LI is a potential tool for assessing disease activity, predicting treatment response, and the overall prognosis in CD through the evaluation of transmural healing [23]. However, further studies are needed to fully validate the use of this index and determine the optimal cut-off values for different patient subgroups.

Preclinical or presymptomatic CD refers to the stage of the disease where there are no symptoms present yet [10]. Diagnosis at this stage is challenging but offers an excellent opportunity to consider interventions that may prevent the symptomatic onset and to better understand the ultimate disease triggers and its pathogenesis. Colorectal cancer screening programs aim to detect colorectal cancer at an early stage when treatment is more likely to be successful. These programs vary by country and region, but most of them target individuals aged 50 years or older, in whom stool and endoscopic examinations, such as colonoscopy or flexible sigmoidoscopy, are performed in an otherwise asymptomatic population. In this context, several cohorts from different countries have reported an approximate 0.35% of new diagnosis of inflammatory bowel disease [8, 24]. Recent data have shown that this pre-disease phase is also associated with a range of altered circulating markers (anti-microbial antibodies [25], anti-GM-CSF [26], anti-integrin αvβ6 [27]), serum protein signatures [28–30], fecal markers [31], increased intestinal permeability [32], proteinuria [33], and dysregulated cellular pathways [28]. In addition, subclinical disease leads to increased healthcare resource utilization and costs, especially related to Primary Care, with more prescriptions of steroids during this period [11, 34, 35]. Hence, it is now clear that the subclinical inflammatory process prior to disease onset has an impact on different aspects, and it is now better characterized.

Despite previous research on early CD and its preclinical disease, this is the first study providing information about the presence of structural damage at this stage. Assessing bowel damage and the subsequent risk of disease progression in CD is important for several reasons. Mainly, it can help predict the risk of complications such as strictures or fistulas and guide treatment decisions. Our study reports new data about cumulative damage along the natural history of the disease, with a focus on early and presymptomatic stages. Here, we were able to describe similar LI scores between subjects with incidental findings of CD and patients with a diagnosis established after the onset of symptoms. This finding suggests that structural bowel damage is already present before the development of overt symptomatic disease. The potential triggers at this point or the threshold for this change still remain unknown. However, our results highlight that among high-risk individuals (e.g., first-degree relatives) [36], certain biomarkers and radiological examinations would be helpful in identifying those with subclinical disease. Early diagnosis would lead to earlier intervention and potentially to disease-modification strategies that would alter the progressive and disabling course of the disease. Despite this potential benefit, we were not able to find predictors of further risk of developing symptoms according to the degree of bowel damage or cross-sectional findings.

Our study also has some limitations that need to be addressed. Its retrospective design with a small number of patients can limit our ability to find significant differences. However, the low prevalence of this type of incidental findings (around 0.35%) should be also considered. In addition, MRE procedures need to have similar protocols and specifications in order to be comparable between patients and centers. Our study used similar procedures, and all examinations were centrally read, so this would have overcome interobserver heterogeneity.

In conclusion, we have shown that the period preceding the symptomatic onset of CD is associated with a significant bowel damage, comparable to those patients with established symptoms. Supporting this observation, our cohort also provides additional data with a description of the main radiological findings that can be found in this clinical setting, confirming that progression towards more chronic changes appear as the disease progresses. This highlights the clear opportunity that the preclinical period creates for future disease intervention strategies that may be able to impact on the natural history of the disease.

## Data Availability

No datasets were generated or analyzed during the current study.
